# A Case of Coexistent Fungal Rhinosinusitis and Actinomycosis Caused by Gutta-Percha Points Extruded Into the Maxillary Sinus

**DOI:** 10.7759/cureus.70246

**Published:** 2024-09-26

**Authors:** Kumiko Terashima, Ryoji Kagoya, Mariko Tanaka, Hironobu Nishijima, Kenji Kondo

**Affiliations:** 1 Department of Otorhinolaryngology and Head and Neck Surgery, Graduate School of Medicine, University of Tokyo, Tokyo, JPN; 2 Department of Pathology, Graduate School of Medicine, University of Tokyo, Tokyo, JPN

**Keywords:** actinomycosis, dental material, fungal ball, gutta-percha points, zinc oxide

## Abstract

Dental materials can stray into the maxillary sinus, causing maxillary sinusitis. We present a case of coexistent fungal rhinosinusitis and actinomycosis caused by gutta-percha points, a core-filling material used for root canal treatment. A 56-year-old man who had undergone root canal treatment with gutta-percha points visited our oral surgery department with complaints of purulent nasal discharge lasting one month. Panoramic radiography confirmed the presence of the foreign body in the left maxillary sinus. As per his wish for tooth preservation, he was referred to our department for the transnasal removal of the material. Nasal endoscopy revealed purulent nasal discharge in the nasal cavity. Computed tomography revealed a soft tissue density lesion filling the left maxillary sinus with a rod-like and spherical radiopaque mass. We performed endoscopic sinus surgery and removed the soft material, in which Candida and actinomycetes were histologically confirmed. Some dental materials containing zinc oxide, such as gutta-percha points, have been reported to cause fungal rhinosinusitis. To our knowledge, there have been no reports of coexisting fungal rhinosinusitis and actinomycosis caused by dental materials extruded into the maxillary sinus. Clinicians should consider the possibility of fungal rhinosinusitis and actinomycosis when removing extruded dental materials.

## Introduction

Dental materials can stray into the maxillary sinus, and maxillary sinusitis caused by the extrusion of dental materials has been previously reported [1]. Gutta-percha, a product derived from tropical rubber plants, is used as a core filling material for root canal treatment [2]. There are many types of core-filling materials, such as zirconium oxide and calcium silicates [3]. Dental material extruded into the sinus is usually removed transorally or transnasally. Some cases of fungal rhinosinusitis caused by extrusion of gutta-percha points into the maxillary sinus have been reported [4-7]. Therefore, surgeons should consider the potential presence of fungal rhinosinusitis when removing the extruded dental material from the maxillary sinus. However, owing to the small number of reports, information on such cases is lacking. Here, we report a rare case of coexistent fungal rhinosinusitis and actinomycosis caused by gutta-percha points extruded into the maxillary sinus.

## Case presentation

**Figure 1:**
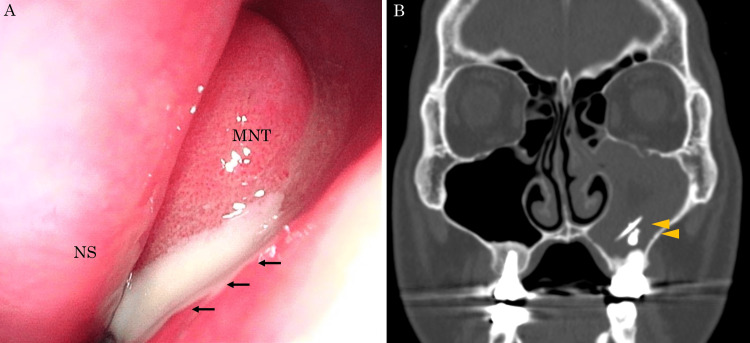
Nasal endoscopy and sinus CT at first visit (A) Endoscopic image of the left nasal cavity. Purulent discharge is observed (arrows). (B) Sinus CT shows a soft tissue density lesion filling the left maxillary sinus with a rod-like and spherical radiopaque mass (arrowheads). CT: computed tomography; MNT: middle nasal turbinate; NS: nasal septum

A 56-year-old man who had undergone root canal treatment of the left upper sixth tooth with gutta-percha points 18 months previously, visited a primary care clinic with complaints of purulent nasal discharge lasting one month. Suspecting maxillary sinusitis caused by the extruded dental material, the patient was referred to the Department of Oral Surgery in our hospital. Panoramic radiography confirmed the presence of a foreign body in the left maxillary sinus, and an oral surgeon suggested extraction of the upper left sixth tooth and transoral removal of the foreign body. However, he strongly desired to preserve his teeth; therefore, he was referred to our department for transnasal removal of the foreign body. Nasal endoscopy revealed purulent nasal discharge draining from the left middle meatus (Figure 1A). A culture of the nasal discharge showed no growth. Plain computed tomography (CT) of the sinuses revealed a soft tissue density lesion filling the left maxillary sinus with a rod-like and spherical radiopaque mass (Figure 1B).

**Figure 2:**
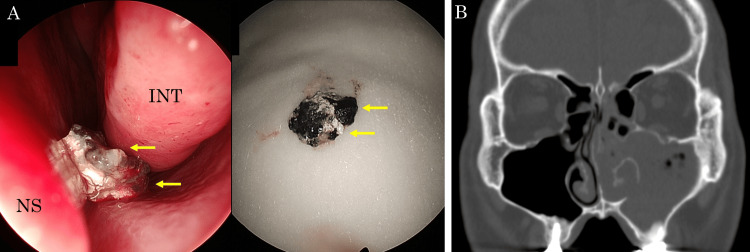
Intraoperative images and sinus CT after surgery (A) Rubber-like hard heterogeneous material flowed out from the maxillary sinus (arrows). (B) Sinus CT on the first postoperative day revealed no radiopaque mass. CT: computed tomography; INT: inferior nasal turbinate; NS: nasal septum

**Figure 3:**
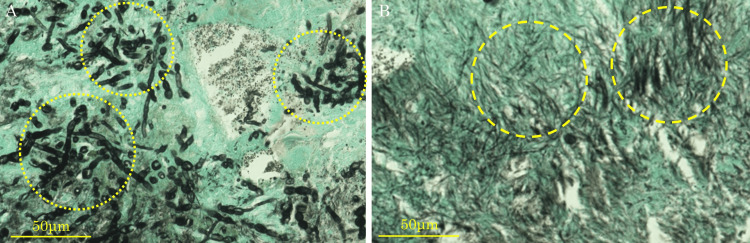
Grocott-stained section of the material (A) Yeast-like organisms with pseudohyphae were observed and diagnosed as Candida (within dotted circles). (B) Grocott stain-positive druse with radially extending hyphae was observed and diagnosed as Actinomyces (within dashed circles).

**Figure 4:**
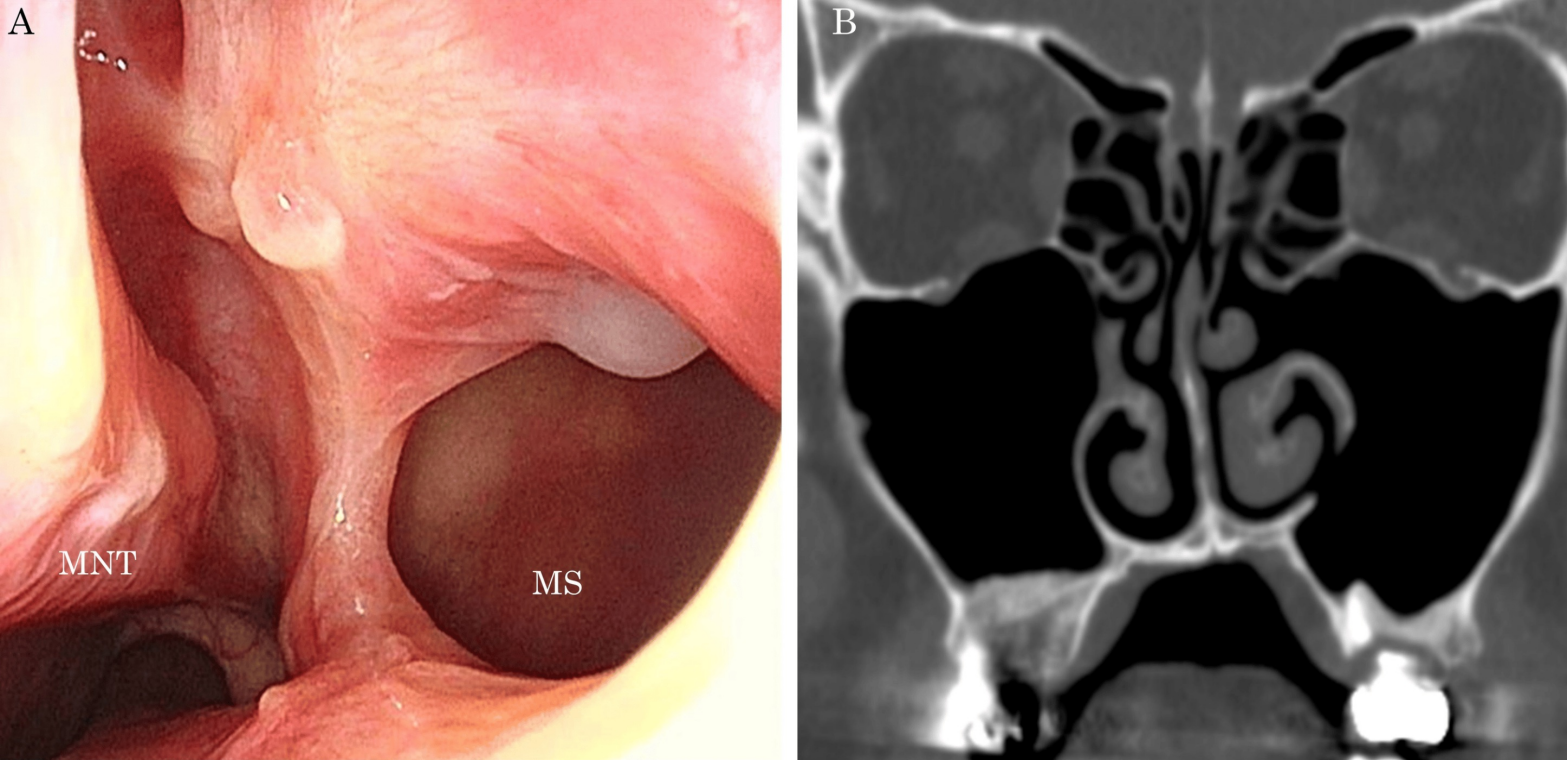
Nasal endoscopy and sinus CT six months after surgery Nasal endoscopy (A) and sinus CT (B) revealed no recurrence of FRS. CT: computed tomography; FRS: fungal rhinosinusitis; MNT: middle nasal turbinate; MS: maxillary sinus

Based on these findings and a history of root canal treatment, the patient was diagnosed with left maxillary sinusitis associated with extruded gutta-percha points. We performed endoscopic sinus surgery (ESS) and transnasal extraction of the gutta-percha points in consultation with the patient. Intraoperatively, we performed maxillary antrostomy and observed drainage of a purulent discharge and soft material 8 mm in size, possibly a foreign body from the maxillary sinus (Figure 2A). The tissues were subjected to a histopathological examination. A control hole was made in the lateral wall of the inferior nasal meatus through which the maxillary sinus was flushed with saline and carefully observed. However, no rod-shaped foreign materials were observed. Sinus CT performed on the first postoperative day revealed the disappearance of the preoperative rod-like radiopaque mass (Figure 2B). A histopathological examination of the resected specimen revealed Candida (Figure 3A) and actinomycetes (Figure 3B). In some areas, fungi that were histologically difficult to distinguish from Aspergillus spp. were also confirmed. At the six-month follow-up, no recurrence of sinusitis was observed without antifungal or antibiotic treatment (Figure 4A, 4B).

## Discussion

Gutta-percha points are core filling materials. They are made of gutta-percha, a type of crude rubber, and are shaped into long, thin points [7]. They contain 66% zinc oxide, 20% gutta-percha, and 11% contrast agent [2]. Core filling materials occasionally stray into the maxillary sinus, particularly when performing procedures on the maxillary first molars, second molars, first premolars, and second premolars [8].

Dental materials containing zinc oxide that extrude into the maxillary sinus can cause fungal rhinosinusitis [9,10]. One of the causes of fungal infections is that zinc is a growth factor for Aspergillus and Candida [11,12]. Another cause is inflammation in the maxillary sinus due to extruded core filling materials, which can disrupt mucociliary transport and prevent the excretion of inhaled Aspergillus spores [9].

To the best of our knowledge, there are only four case reports of fungal rhinosinusitis caused by root canal-filling materials, such as gutta-percha points [4-7]. Although few cases have been reported, the actual number of cases may be much larger because zinc oxide is a fungal nourishment, as mentioned previously [9,10]. When removing a foreign body extruded into the maxillary sinus, it is essential to carefully evaluate preoperative images and observe the maxillary sinus perioperatively to avoid overlooking fungal infections.

In this case, the resected material was pathologically diagnosed as Candida species. There have been no previous reports of fungal rhinosinusitis caused by core-filling materials in which Candida spp. have been detected. Although coexistent fungal ball and actinomycosis have been reported [13,14], there have been no reports of actinomycosis caused by extruded dental materials. It is not always easy to observe the entire maxillary sinus intranasally; therefore, surgeons should consider the possibility of fungi or actinomycetes as well as foreign bodies in the maxillary sinus when extracting dental material. Generally, surgical removal of the lesion and postoperative antimicrobial treatment are recommended in cases of actinomycosis [15]. In this case, we achieved maxillary sinus drainage and foreign body removal, and there were no intraoperative findings suggestive of submucosal infiltration of Actinomyces or fungi. Therefore, we chose careful follow-up without postoperative antibiotic therapy.

One limitation of the present case is that the rod-like foreign body confirmed by preoperative CT could not be identified intraoperatively. One possible reason for this is that it was aspirated intraoperatively despite careful manipulation. Another possible reason is that it was deformed by thermal degeneration after preoperative CT, which was performed three months before surgery. In addition, the rod-like lesion could also have been a calcified lesion associated with fungal rhinosinusitis.

## Conclusions

We report a rare case of coexistent fungal rhinosinusitis and actinomycosis caused by gutta-percha points extruded into the maxillary sinus. The present case adds to the current knowledge by emphasizing that core-filling materials containing zinc oxide can cause fungal rhinosinusitis and actinomycosis. Clinicians should consider this possibility when removing extruded dental materials.
